# Association between surgical procedures under general anesthesia in infancy and developmental outcomes at 1 year: the Japan Environment and Children’s Study

**DOI:** 10.1186/s12199-020-00873-6

**Published:** 2020-07-25

**Authors:** Yoshiko Kobayashi, Narumi Tokuda, Sho Adachi, Yasuhiro Takeshima, Munetaka Hirose, Masayuki Shima, Japan Environment and Children’s Study (JECS) Group, Michihiro Kamijima, Shin Yamazaki, Yukihiro Ohya, Reiko Kishi, Nobuo Yaegashi, Koichi Hashimoto, Chisato Mori, Shuichi Ito, Zentaro Yamagata, Hidekuni Inadera, Takeo Nakayama, Hiroyasu Iso, Youichi Kurozawa, Narufumi Suganuma, Koichi Kusuhara, Takahiko Katoh

**Affiliations:** 1 Hyogo Regional Center for the Japan Environment and Children’s Study, Hyogo College of Medicine, Nishinomiya, Japan; 2 Department of Anesthesiology and Pain Medicine, Hyogo College of Medicine, Nishinomiya, Japan; 3 Department of Public Health, Hyogo College of Medicine, Mukogawa-cho, Nishinomiya, Hyogo 663-8501 Japan; 4 Department of Pediatrics, Hyogo College of Medicine, Nishinomiya, Japan

**Keywords:** Infant, Development, Surgery, General anesthesia, Neurotoxicity, Ages and Stages Questionnaires (ASQ), Japan Environment and Children’s Study (JECS)

## Abstract

**Background:**

The neurotoxicity of general anesthesia to the developing human brains is controversial. We assessed the associations between surgery under general anesthesia in infancy and development at age 1 year using the Japan Environment and Children’s Study (JECS), a large-scale birth cohort study.

**Methods:**

In the JECS, 103,062 pregnancies and 104,065 fetuses were enrolled between January 2011 and March 2014. Of the 100,144 registered live births, we excluded preterm or post-term infants, multiple births, and infants with chromosomal anomalies and/or anomalies of the head or brain. Data on surgical procedures under general anesthesia in infancy were collected from self-administered questionnaires by parents at the 1-year follow-up. Developmental delay at age 1 year was assessed using the Japanese translation of the Ages and Stages Questionnaires, Third Edition (J-ASQ-3), comprising five developmental domains.

**Results:**

Among the 64,141 infants included, 746 infants had surgery under general anesthesia once, 90 twice, and 71 three or more times. The percentage of developmental delay in the five domains of the J-ASQ-3 significantly increased with the number of surgical procedures. After adjusting for potential confounding factors, the risk of developmental delays in all five domains was significantly increased in infants who had surgery under general anesthesia three times or more (adjusted odds ratios: for communication domain 3.32; gross motor domain 4.69; fine motor domain 2.99; problem solving domain 2.47; personal–social domain 2.55).

**Conclusions:**

Surgery under general anesthesia in infancy was associated with an increased likelihood of developmental delay in all five domains of the J-ASQ-3, especially the gross motor domain at age 1 year. The neurodevelopment with the growth should be further evaluated among the children who had surgery under general anesthesia.

**Trial registration:**

UMIN Clinical Trials Registry (number: UMIN000030786)

## Introduction

Recently, the influence of anesthesia on children’s neurodevelopment has attracted attention. Numerous animal studies have indicated that exposing the developing brain to anesthetics may lead to widespread apoptotic neurodegeneration, interference with synaptogenesis during brain development, and long-term neurocognitive impairment [1–6]. However, the detailed mechanism has not been clarified. Results in human children, however, remain controversial. Three large-scale prospective studies, the General Anesthesia compared to Spinal anesthesia (GAS) trial [7, 8], the Pediatric Anesthesia Neurodevelopment Assessment (PANDA) study [9], and the Mayo Anesthesia Safety in Kids (MASK) study [10], demonstrated that a single, relatively short exposure to general anesthesia in infants and toddlers is unlikely to cause clinically detectable neurodevelopmental deficits or serious behavior disorders at age 5 years, 8–15 years, and 8–12 or 15–20 years, respectively. However, the effects of repeated and prolonged anesthetic exposure in children are unknown. The US Food and Drug Administration (FDA) warned that repeated or lengthy use of general anesthetics in children younger than 3 years of age may affect brain development [11, 12].

Although numerous studies have been conducted, there is no large-scale Japanese study investigating the neurotoxicity of anesthesia. The Japan Environment and Children’s Study (JECS) is an ongoing prospective nationwide birth cohort study in Japan, and the participating children will be followed up until they reach 13 years of age. In this study, we investigated the association of surgery under general anesthesia in infancy with developmental outcomes at age 1 year using data from the JECS.

## Materials and methods

### Design and subjects

The data were obtained from the ongoing JECS, for which pregnant women were recruited between January 2011 and March 2014 from 15 regions throughout Japan. The JECS was designed to investigate mothers and children until the latter is 13 years old. Scheduled to continue until 2027, the JECS measures the effect of environmental factors on children’s health. This study was registered in the UMIN Clinical Trials Registry (number: UMIN000030786). The details of the JECS have been described elsewhere [13, 14]. The JECS protocol was approved by the Institutional Review Board on Epidemiological Studies of the Ministry of the Environment and the review boards of all participating institutions. The JECS is conducted in accordance with the Declaration of Helsinki and other internationally recognized regulations. Written informed consent was obtained from all participants.

After registration, the women answered self-administered questionnaires twice: in the first trimester and the second/third trimester. The father’s information was also collected using a self-administered questionnaire at a time between the mother’s recruitment and the child’s first-month check-up. Children’s medical records were transcribed by physicians, midwives, nurses, and/or research coordinators after delivery, and 1 month after birth. Children were followed up mainly through self-administered questionnaires completed by their mothers or the mothers’ partners at 1 month, 6 months, and 1 year after birth.

This study is based on the “jecs-an-20180131” dataset, released in March 2018. The JECS enrolled 103,062 pregnancies, 104,065 fetuses, and 100,144 live births. Among the live births, we excluded preterm (< 37 weeks) or post-term (> 41 weeks) infants, multiple births, and infants with congenital diseases, all of which can affect development. The information on gestational age and multiple births were collected using medical record transcripts at birth. The congenital diseases excluded were chromosomal anomalies and anomalies of the head or brain. Information regarding chromosomal anomalies (such as Down syndrome, trisomy 18, trisomy 13, and Turner syndrome) was collected from medical records at the 1-month check-up and self-administered questionnaires at 1 year. Information regarding anomalies of the head or brain (such as anencephaly, encephalocele, microcephaly, hydrocephalus, craniotabes, holoprosencephaly, and agenesis of the corpus callosum) was obtained from medical records at the first-month check-up. Information on whether the child underwent surgery under general anesthesia before age 1 year was obtained from the question “Number of surgical procedures under general anesthesia” in the questionnaire administered at the 1-year follow-up. This questionnaire did not cover the date of surgery under general anesthesia, the disease that necessitated the procedure, surgery name, or anesthesia type.

The Ages and Stages Questionnaire-Third Edition (ASQ-3) is a parent-completed developmental screening measure whose utility has been demonstrated [15–18]. This standardized tool for infants and children aged 1–66 months includes 30 questions across five developmental domains: communication, gross motor, fine motor, problem solving, and personal–social. The response options “yes,” “sometimes,” or “not yet,” scored 10, 5, and 0, respectively, and the total points for each domain are calculated. For each domain, a cutoff score is determined by subtracting two standard deviations from the mean for each applied month; if the score is less than the cutoff, the child is evaluated as “requiring a referral for further assessment.” We used the Japanese translation of the ASQ-3 (J-ASQ-3) [19] for the evaluation of developmental outcomes at age 1 year, but the cutoff score was taken from the original ASQ-3 because its psychometric profile has been validated. The details of the ASQ-3 were provided by Squires and Bricker [20].

**Fig. 1:**
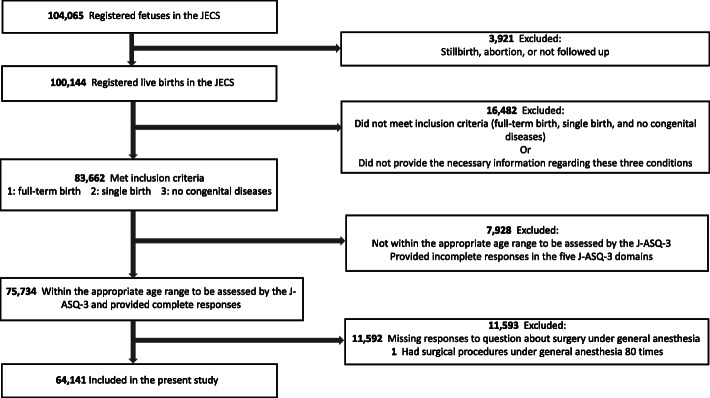
Flowchart of subject selection. Of the 104,065 fetuses that were registered in the JECS, stillbirths, abortions, and those who were not followed up were excluded, and 100,144 live births were finally registered. Then, 83,662 infants who met the following conditions—full-term birth, single birth, and no congenital disease—were considered. The congenital diseases excluded were chromosomal anomalies and anomalies of the head or brain. After excluding infants with missing data regarding surgery under general anesthesia or any J-ASQ-3 domains and who were not within the applicable assessment age for the J-ASQ-3, the final study population included 64,141 infants

Infants with missing information regarding gestational age, pregnancy outcome, chromosomal anomalies, anomalies of the head or brain, number of surgical procedures under general anesthesia, or J-ASQ-3 results were excluded. Thus, the 64,141 infants remained to be included in our analysis. The subject selection process is depicted in Fig. 1.

### Statistical analysis

We examined the association between surgery under general anesthesia in infancy and development at age 1 year. We divided the subjects into four groups, depending on the number of surgical procedures under general anesthesia: none, once, twice, and three times or more. Infants below and above the cutoff for each domain were termed “delayed” and “normal,” respectively. In univariate analysis, the association between the delay in each J-ASQ-3 domain and the number of surgical procedures under general anesthesia was evaluated using the Cochran–Armitage test. Subsequently, multiple logistic regression analyses were performed adjusting for confounders (sex, gestational age at birth, birth weight, 5-min Apgar score, delivery method, maternal age at birth, presence of siblings, and underlying congenital diseases at age 1 year). The information regarding sex, gestational age, birth weight, 5-min Apgar score, delivery method, and maternal age at birth was collected from the medical record transcription at birth, while information on congenital diseases was obtained from the self-administered questionnaires at 1 year of age. For sensitivity analyses, the models were re-run using maternal age as a categorical variable. Additional analyses adjusting for paternal age at registration, parents’ educational level, and household income were conducted. Besides, we also conducted the analyses excluded infants with congenital heart diseases, because infants who had such diseases are known to be at high risk for developmental delay [21]. Furthermore, we conducted the analyses using the cutoff score of each J-ASQ-3 domain reported for Japanese children [19]. Information regarding paternal age at registration, presence of siblings, parents’ educational level, and household income was collected from the parents’ self-administered questionnaires.

A *p* value of < 0.05 was considered statistically significant. Multiple logistic regression analyses were performed using SPSS Statistics ver. 24 (IBM), and the Cochran–Armitage test was performed using the R software ver. 3.5.0.

## Results

Subject characteristics are shown in Table 1. Among the 64,141 infants included, 746 (1.2%) had surgery under general anesthesia once, 90 (0.1%) had twice, and 71 (0.1%) had three times or more.

**Table 1:** Characteristics of the study subjects

	All subjects (*N* = 64,141)
Sex of infants	
Boy	32,727 (51.0%)
Girl	31,414 (49.0%)
Gestational age at birth, mean [SD], weeks	39.1 [1.1]
Birth weight, g	
< 2000	115 (0.2%)
2000–2499	3268 (5.1%)
2500–3999	60,186 (93.8%)
≥ 4000	558 (0.9%)
Missing	14 (0.0%)
Apgar score at 5 min	
0–3	68 (0.1%)
4–6	127 (0.2%)
≥ 7	60,890 (94.9%)
Missing	3056 (4.8%)
Delivery method	
Vaginal	52,974 (82.6%)
Caesarean section	11,022 (17.2%)
Missing	145 (0.2%)
Maternal age at birth, mean [SD], years	31.3 [4.9]
< 20	368 (0.6%)
20–24	5002 (7.8%)
25–34	41,294 (64.4%)
35–39	14,617 (22.8%)
≥ 40	2857 (4.5%)
Missing	3 (0.0%)
Number of siblings	
0	29,565 (46.1%)
1	23,509 (36.7%)
≥ 2	10,756 (16.8%)
Missing	311 (0.5%)
Number of surgical procedures under general anesthesia	
0	63,234 (98.6%)
1	746 (1.2%)
2	90 (0.1%)
≥ 3	71 (0.1%)
Congenital disease at age 1	
Head and face	
Yes	217 (0.3%)
No	63,924 (99.7%)
Eye and ear	
Yes	506 (0.8%)
No	63,635 (99.2%)
Spinal cord	
Yes	52 (0.1%)
No	64,089 (99.9%)
Heart	
Yes	865 (1.3%)
No	63,276 (98.7%)
Chest (without heart) and digestive organs (abdomen)	
Yes	339 (0.5%)
No	63,802 (99.5%)
Skin	
Yes	1378 (2.1%)
No	62,763 (97.9%)
Upper and lower limb/muscle/bone/joint	
Yes	412 (0.6%)
No	63,729 (99.4%)
Kidney/urogenital apparatus	
Yes	562 (0.9%)
No	63,579 (99.1%)
Endocrine system/hormone	
Yes	130 (0.2%)
No	64,011 (99.8%)
Metabolism	
Yes	20 (0.0%)
No	64,121 (100.0%)
Others	
Yes	229 (0.4%)
No	63,912 (99.6%)

Data are *n* (%) unless otherwise specified

*SD* standard deviation

Table 2 shows the cutoff scores at 12 months for each domain of the J-ASQ-3 and the numbers and percentages of normal and delayed infants. The percentage of infants with communication delays was the lowest (7.0%), while personal–social delays were the highest (16.8%).

**Table 2:** Original cutoff scores of each J-ASQ-3 domain and numbers of normal and delayed infants

	Original cutoff score at 12 months	All subjects (*N* = 64,141)	
		Above cutoff score (normal infants)	Below cutoff score (delayed infants)
Communication	15.64	59,679 (93.0%)	4462 (7.0%)
Gross motor	21.49	55,217 (86.1%)	8924 (13.9%)
Fine motor	34.50	57,838 (90.2%)	6303 (9.8%)
Problem solving	27.32	54,316 (84.7%)	9825 (15.3%)
Personal–social	21.73	53,373 (83.2%)	10,768 (16.8%)

Data are *n* (%) unless otherwise specified. The cutoff scores were taken from the original ASQ-3 [20]

*J-ASQ-3* Japanese translation of the Ages and Stages Questionnaire-Third Edition

Table 3 shows the numbers and percentages of infants with delays for each domain by the number of surgical procedures under general anesthesia. Among infants who had surgery under general anesthesia more than once, the percentages of delays were high in all domains. The tendency toward developmental delay in all five domains with increasing number of surgical procedures was recognized by the Cochran–Armitage test (*p* < 0.001 in all domains).

**Table 3:** Number of delayed infants for each J-ASQ-3 domain, according to the number of surgery

J-ASQ-3	Surgery under general anesthesia				*p* value for trend^a^
	None (*n* = 63,234)	1 time (*n* = 746)	2 times (*n* = 90)	≥ 3 times (*n* = 71)	
Communication	4373 (6.9%)	64 (8.6%)	9 (10.0%)	16 (22.5%)	< 0.001
Gross motor	8695 (13.8%)	159 (21.3%)	36 (40.0%)	34 (47.9%)	< 0.001
Fine motor	6167 (9.8%)	94 (12.6%)	23 (25.6%)	19 (26.8%)	< 0.001
Problem solving	9628 (15.2%)	145 (19.4%)	27 (30.0%)	25 (35.2%)	< 0.001
Personal–social	10,558 (16.7%)	151 (20.2%)	32 (35.6%)	27 (38.0%)	< 0.001

Data are *n* (%) unless otherwise specified. The cutoff scores from the original ASQ-3 were used [20]

*J-ASQ-3* Japanese translation of the Ages and Stages Questionnaire-Third Edition

^a^Cochran–Armitage test

**Fig. 2:**
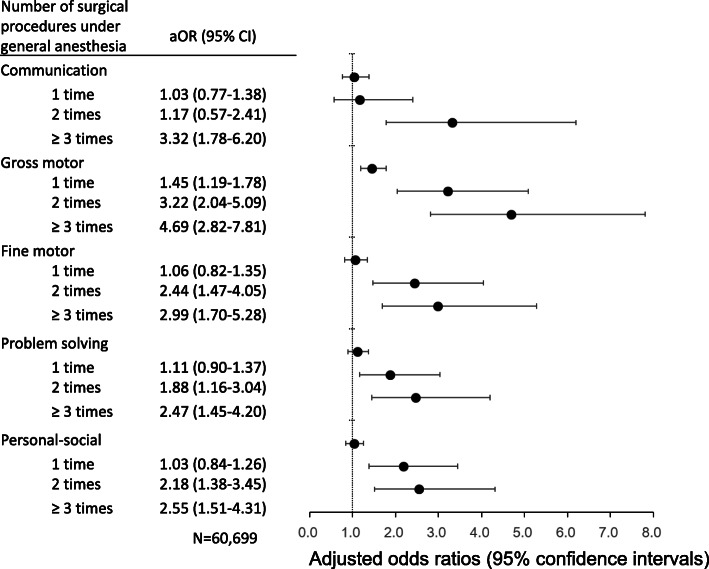
Adjusted odds ratios of delayed infants among infants who had surgical procedures under general anesthesia compared with infants who did not have surgery, according to the number of surgical procedures under general anesthesia for each of the five domains. aOR adjusted odds ratio, CI confidence interval. Adjusted for sex, gestational age, birth weight, Apgar score at 5 min, delivery method, maternal age at birth, presence of siblings, and presence of congenital disease, compared with infants who did not have surgery under general anesthesia. The cutoff scores from the original ASQ-3 were used

Figure 2 depicts the adjusted odds ratios (aORs) obtained from logistic analyses for all five domains. The aORs of gross motor delay in infants who had surgical procedures under general anesthesia compared with those who did not have surgery were 4.69 [95% confidence interval (CI) 2.82–7.81] for three or more procedures, 3.22 [95% CI 2.04–5.09] for two procedures, and 1.45 [95% CI 1.19–1.78] for one procedure. For the fine motor, problem solving, and personal–social domains, the aORs of developmental delays were significantly increased in infants who had surgery under general anesthesia three times or more (2.99 [95% CI 1.70–5.28], 2.47 [95% CI 1.45–4.20], and 2.55 [95% CI 1.51–4.31], respectively). In these three domains, the aORs of developmental delay were significantly higher among infants exposed to surgery twice than among unexposed infants, but this was not the case with infants exposed once. For the communication domain, infants exposed to surgery three times or more had a significantly higher risk than unexposed infants did (aOR 3.32 [95% CI 1.78–6.20]). In all five domains, the risk of developmental delays was increased with three or more surgical procedures under general anesthesia.

The above analyses were conducted using maternal age as a continuous variable. When it was used as a categorical variable, the results were much the same (Additional file 1). When paternal age, parents’ educational level, and household income were additionally adjusted, the risks in the infants exposed to surgery were elevated, although the number of subjects decreased (from 64,141 to 30,448) owing to the limited number of registered fathers (Additional file 2). In the additional analyses after excluding infants who had congenital heart diseases, the aORs of developmental delays were also increased among infants who had surgery under general anesthesia twice or more (Additional file 3). In the analyses using the cutoff scores of the J-ASQ-3 reported for Japanese children, the results were approximately similar, and the aORs were generally higher (Additional files 4, 5, 6).

## Discussion

Recently, the neurotoxicity of general anesthesia to the developing brains has been a topic of concern. In the JECS study, the participating children will be followed up until they reach 13 years of age. In the present study, we evaluated whether surgery under general anesthesia in infancy was associated with developmental delay, using data from the JECS. A large percentage of infants who had surgery under anesthetic exposure displayed developmental delays in all five J-ASQ-3 domains at age 1 year, and this percentage increased significantly with the number of surgical procedures under general anesthesia. In multivariate analysis, the risk of developmental delays in all five J-ASQ-3 domains increased when the surgical procedures under general anesthesia numbered three or more. Gross motor development was the most affected, and the risk of the gross motor delay was increased by exposure to surgery even once.

Many studies have investigated the association between general anesthetic exposure and adverse neurodevelopmental outcomes in human children. Of the prospective studies, the GAS trial [7, 8] was an international, multicenter, randomized, controlled, equivalence trial. A total of 722 infants under 60 weeks’ postmenstrual age, who were undergoing inguinal hernia surgery, were randomly assigned to one of two anesthetic techniques: general anesthesia with inhaled sevoflurane or awake regional anesthesia (with no general anesthesia exposure). The intelligence quotient (IQ) at age 5 years [7] and neurodevelopmental outcomes at age 2 years [8] were assessed using the Bayley Scales of Infant and Toddler Development, Third Edition (BSID-III) [22]; no significant differences in the primary outcomes were found between the two groups. The PANDA study [9], a multicenter, ambidirectional observational study, examined a sibling cohort discordant for exposure to general anesthesia for inguinal hernia repair. The sibling pairs were under 36 months of age. There was no statistically significant difference in the IQ scores between sibling pairs at age 8–15 years. Despite their contributions to the literature, GAS and PANDA did not clearly elucidate the risks of repeated and prolonged anesthetic exposure. In the MASK study [10], an ambidirectional observational study, children who were unexposed, singly exposed, or multiply exposed to anesthesia before age 3 years underwent neuropsychological testing at age 8–12 years, or age 15–20 years. Although the IQ did not differ by exposure status, it was suggested that multiple, but not single, exposures were associated with a pattern of changes in specific neuropsychological domains associated with behavioral and learning difficulties.

Various retrospective studies have also investigated the association between general anesthesia in childhood and adverse neurodevelopmental outcomes. While some have concluded that there is no association [23–31], others have suggested otherwise [21, 32–42]. Among the studies reporting that anesthesia does not affect development, many have either set the timing of developmental evaluation at adolescence [23–26], early after surgery [27, 28], or have considered the period before the age of 3–6 years as the time of exposure to anesthesia [23, 24, 27–30]. However, there are also studies considering the effects of anesthesia received in infancy that are similar to our study [25, 26]. Hansen et al. [25] reported that single, relatively brief anesthetic exposure in connection with hernia repair in infancy did not reduce academic performance at age 15 or 16 years, after adjusting for known confounders. In studies of anesthesia and development, a variety of exposure periods and outcomes has been reported, including neuropsychological assessment [21, 32–35], degree of learning disability [36–39], and academic performance [40, 41]. Some described the effects of anesthesia exposure in infancy [21, 34, 40, 42], while some had multiple anesthesia exposures [38, 39]. Wilder et al. [39] reported that a single exposure to anesthesia by age 4 years was not associated with an increased risk of learning disability, but those with greater exposure were at an increased risk of a learning disability (hazard ratios = 1.59 for two anesthetics and 2.60 for three or more anesthetics). The MASK study [10] reevaluated the same topic using an ambidirectional design. Despite controversial results and a lack of consensus on the effects of multiple and long-term anesthesia exposures, the results of the GAS trial, MASK study, and PANDA study suggest that a single and brief exposure to anesthesia may not be associated with neurodevelopmental disorders and behavioral problems [7–12, 43].

Our study demonstrates that surgery under general anesthesia in infancy is associated with developmental delay, as assessed by the J-ASQ-3 method at age 1 year. The timing of the outcome evaluation, however, is important due to the plasticity of neural development in early life: most studies reporting that anesthesia has no influence tend to evaluate development several months after surgery or even in adolescence. We evaluated development at age 1 year, which is relatively early. The JECS conducts follow-ups until age 13 years, and so, there is an opportunity to follow changes in this cohort over a long period. When evaluating neurotoxicity due to anesthesia, it is important to select an outcome measure that can comprehensively evaluate development [43–45]. Reviewing studies according to outcome measure, Davidson et al. [43] found that academic performance or school readiness tests are not greatly affected by anesthesia exposure in early life. Furthermore, results were inconsistent when abnormalities in neurocognitive function or behavior (based on validated neuropsychological or behavior assessment tools) were the outcome. The J-ASQ-3 is a developmental assessment tool and may lead to inconsistent outcomes.

Adverse developmental outcomes increased with the number of anesthetic exposures in previous studies, which our study confirmed [38, 39]. Also, two studies have used the BSID-III [22] to evaluate development at age 1 year among children exposed to anesthesia in infancy [21, 34]. Walker et al. [21] reported that infants who underwent cardiac surgery within 90 days of birth had significantly lower mean scores in all five domains of the BSID-III, and non-cardiac major surgery mean scores were lower in four domains compared with controls. Although the outcomes used differed from our study, the results were similar. Furthermore, it is interesting to note that gross motor delay was most affected in both the cardiac and non-cardiac surgery groups in Walker et al.’s study [21] and our study. On the contrary, Ing et al. [32] reported that motor function was not impaired among children exposed to anesthesia before age 3 years. Furthermore, in the MASK study [10], processing speed and fine motor abilities were decreased in children after multiple anesthetic exposures. Thus, there is no consensus regarding which domain is most susceptible to anesthesia, and further studies are needed. In our study, we used the cutoff scores of the original ASQ-3, but the cutoff scores of the J-ASQ-3 for Japanese children were published only recently. The cutoff scores reported for Japanese children were considerably lower than original scores, particularly for the communication and personal–social domains. When we also conducted a sensitivity analysis using the Japanese cutoff score (Additional files 4, 5, 6), the results were approximately similar, and the aORs were generally higher.

The main strength of our study was that we could examine children who had surgery under general anesthesia in a large-scale birth cohort study, including the 64,141 infants. However, our study had some limitations. First, information on surgery under general anesthesia was obtained from the mothers’ questionnaire responses, not medical records, and we did not confirm their records with the medical institution. In addition, details such as time and type of surgery and anesthesia were unknown. Second, when the subjects were selected, chromosomal anomalies and anomalies of the head or brain were the only congenital diseases excluded. Therefore, diseases that required multiple operations and long-term and multiple hospitalizations, such as congenital heart disease and digestive system disease, might have been inadvertently included. Considering the developmental effects of these diseases themselves and the increases in hospitalization they lead to, it is difficult to say that our findings are purely related to general anesthesia. Third, developmental outcomes were evaluated only at age 1 year. Individual differences in infancy are large, and it is unclear to what degree developmental delays at age 1 year can predict future prognosis. In addition, some researchers have questioned the accuracy of the ASQ-3 in children less than 2 years of age [46, 47]. Fourth, the associations of developmental delays with surgery under general anesthesia were analyzed in a cross-sectional manner because information on both of the surgical procedures and the responses to the J-ASQ were obtained from the 1-year questionnaire. However, our study is based on a birth cohort study, and the children will be followed up until age 13 years. Therefore, the association between surgery under general anesthesia in infancy and developmental outcomes can be further elucidated.

Here, we would like to emphasize that general anesthetic and sedation drugs are essential for young children who require surgery or other painful and stressful procedures. Some clinical studies have demonstrated that inadequate sedation, anesthesia, and analgesia may actually increase the risk of adverse postoperative outcomes in infants and children [48–52]. Therefore, as recommended by the FDA [11], concerns about anesthesia affecting the developing brain should not stand in the way of necessary surgeries or procedures. At this stage, it is necessary to balance the benefits of appropriate anesthesia in young children with the potential risks, especially for procedures that may last longer than 3 h or in the case of children who require multiple procedures before age 3 years [11, 12]. If surgical procedures under general anesthesia in infancy are required, we think that the treatment should be performed with the short and minimum times of anesthesia. In addition, it is desirable that the neurodevelopment with the growth of the child will be followed up after surgery.

## Conclusions

We investigated whether surgery under general anesthesia in infancy was associated with developmental delays at age 1 year using the JECS data. A significantly high percentage of infants who had surgery under general anesthesia displayed delays in all five J-ASQ-3 domains, and there was a tendency toward greater development delays as the number of surgical procedures increased. In all the five domains, the risk of developmental delays may increase among infants who had surgery under general anesthesia three times or more, especially in the gross motor domain. However, follow-up studies are essential to confirm the results and to elucidate whether the observed delays persist, worsen, or improve as the children grow. At this stage, the balance of the benefits of appropriate anesthesia in young children with the potential risks should be considered.

## Supplementary information

**Additional file 1. **Adjusted odds ratios of developmental delay among infants who had surgical procedures under general anesthesia compared with infants who did not have surgery, for each of the five domains (maternal age analyzed as categorical variable) (*N* = 60,699)

**Additional file 2. **Adjusted odds ratios of developmental delay among infants who had surgical procedures under general anesthesia compared with infants who did not have surgery, for each of the five domains (analysis included paternal age at registration, parents’ educational level, and household income) (*N* = 30,448)

**Additional file 3. **Adjusted odds ratios of developmental delay among infants who had surgical procedures under general anesthesia compared with infants who did not have surgery, for each of the five domains (analysis excluded infants with congenital heart disease) (*N* = 60,310)

**Additional file 4.** Cutoff scores of each J-ASQ-3 domain reported for Japanese children, and numbers of normal and delayed infants

**Additional file 5.** Number of delayed infants for each J-ASQ-3 domain, according to the number of surgery under general anesthesia using the cutoff scores reported for Japanese children

**Additional file 6. **Adjusted odds ratios of developmental delay among infants who had surgical procedures under general anesthesia compared with infants who did not have surgery, for each of the five domains using the cutoff scores of the J-ASQ-3 reported for Japanese children (*N* = 60,699)

## Data Availability

The data that support the findings of this study are unsuitable for public deposition due to ethical restrictions and specific legal framework in Japan. It is prohibited by the Act on the Protection of Personal Information (Act No. 57 of 30 May 2003, amended on 9 September 2015) to publicly deposit data containing personal information. The Ethical Guidelines for Epidemiological Research enforced by the Japan Ministry of Education, Culture, Sports, Science, and Technology and the Ministry of Health, Labor, and Welfare also restricts the open sharing of the epidemiologic data.

## Abbreviations

BSID-III: The Bayley Scales of Infant and Toddler Development, Third Edition
IQ: Intelligence quotient
ASQ-3: The Ages and Stages Questionnaire-Third Edition
J-ASQ-3: The Japanese translation of the ASQ-3
JECS: The Japan Environment and Children’ Study
FDA: The US Food and Drug Administration
aOR: Adjusted odds ratio
CI: Confidence interval
GAS trial: The General Anesthesia compared to Spinal anesthesia trial
PANDA study: The Pediatric Anesthesia Neurodevelopment Assessment study
MASK study: The Mayo Anesthesia Safety in Kids study
