# Supplementary material for: Association between surgical procedures under general anesthesia in infancy and developmental outcomes at 1 year: the Japan Environment and Children’s Study
Source: Environ Health Prev Med. 2020 Jul 25;25:32. doi: 10.1186/s12199-020-00873-6 (PMC7382792; doi:10.1186/s12199-020-00873-6)
Supplement: Supplementary file 1 — Additional file 1. Adjusted odds ratios of developmental delay among infants who had surgical procedures under general anesthesia compared with infants who did not have surgery, for each of the five domains (maternal age analyzed as categorical variable) (N = 60,699) [file 12199_2020_873_MOESM1_ESM.docx]

**Additional file 1** Adjusted odds ratios of developmental delay among infants who had surgical procedures under general anesthesia compared with infants who did not have surgery, for each of the five domains (maternal age analyzed as categorical variable) (N=60,699).

|  | Surgery under general anesthesia | | |
| --- | --- | --- | --- |
| J-ASQ-3 | 1 time | 2 times | ≥3 times |
|  | aOR (95% CI) | aOR (95% CI) | aOR (95% CI) |
| Communication | 1.04 (0.77-1.39) | 1.15 (0.56-2.37) | 3.31 (1.77-6.17) |
| Gross motor | 1.46 (1.19-1.79) | 3.16 (2.00-4.98) | 4.64 (2.79-7.72) |
| Fine motor | 1.06 (0.83-1.36) | 2.43 (1.46-4.03) | 2.97 (1.68-5.23) |
| Problem solving | 1.11 (0.90-1.37) | 1.87 (1.16-3.01) | 2.44 (1.43-4.14) |
| Personal-social | 1.03 (0.84-1.27) | 2.16 (1.36-3.42) | 2.53 (1.50-4.28) |

Abbreviations: aOR, adjusted odds ratio; CI, confidence interval; J-ASQ-3, Japanese translation of the Ages and Stages Questionnaire-Third Edition.

Adjusted for sex, gestational age, birth weight, Apgar score at five minutes, delivery method, maternal age at birth, presence of siblings, and presence of congenital disease, compared with infants who did not have surgery. Categories of maternal age: <20 years, 20-24 years , 25-34 years, 35-39 years, ≥40 years

The cutoff scores from the original ASQ-3 were used [20].
