# Supplementary material for: Association between surgical procedures under general anesthesia in infancy and developmental outcomes at 1 year: the Japan Environment and Children’s Study
Source: Environ Health Prev Med. 2020 Jul 25;25:32. doi: 10.1186/s12199-020-00873-6 (PMC7382792; doi:10.1186/s12199-020-00873-6)
Supplement: Supplementary file 4 — Additional file 4. Cutoff scores of each J-ASQ-3 domain reported for Japanese children, and numbers of normal and delayed infants [file 12199_2020_873_MOESM4_ESM.docx]

**Additional file 4** Cutoff scores of each J-ASQ-3 domain reported for Japanese children, and numbers of normal and delayed infants

| J-ASQ-3 | Cutoff score at 12 months* | All subjects （N=64,141） | |
| --- | --- | --- | --- |
|  |  | Above cutoff score（normal infants） | Below cutoff score（delayed infants） |
| Communication | 4.53 | 64,068 (99.9%) | 73 (0.1%) |
| Gross motor | 9.43 | 60,584 (94.5%) | 3,557 (5.5%) |
| Fine motor | 25.47 | 60,464 (94.3%) | 3,677 (5.7%) |
| Problem solving | 15.37 | 60,903 (95.0%) | 3,238 (5.0%) |
| Personal-social | 4.95 | 63,428 (98.9%) | 713 (1.1%) |

Data are n (%) unless otherwise specified.

Abbreviations: J-ASQ-3, Japanese translation of the Ages and Stages Questionnaire-Third Edition.

* The cutoff scores of the J-ASQ-3 reported for Japanese children were used [19].
