# Supplementary material for: Association between surgical procedures under general anesthesia in infancy and developmental outcomes at 1 year: the Japan Environment and Children’s Study
Source: Environ Health Prev Med. 2020 Jul 25;25:32. doi: 10.1186/s12199-020-00873-6 (PMC7382792; doi:10.1186/s12199-020-00873-6)
Supplement: Supplementary file 5 — Additional file 5. Number of delayed infants for each J-ASQ-3 domain, according to the number of surgery under general anesthesia using the cutoff scores reported for Japanese children [file 12199_2020_873_MOESM5_ESM.docx]

**Additional file 5** Number of delayed infants for each J-ASQ-3 domain, according to the number of surgery under general anesthesia using the cutoff scores reported for Japanese children

| J-ASQ-3 | Surgery under general anesthesia | | | | P value for trend^†^ |
| --- | --- | --- | --- | --- | --- |
|  | None  (n=63,234) | 1 time  (n=746) | 2 times  (n=90) | ≥3 times  (n=71) |  |
| Communication | 64 (0.1%) | 2 (0.3%) | 0 (0.0%) | 7 (9.9%) | <0.001 |
| Gross motor | 3,419 (5.4%) | 84 (11.3%) | 28 (31.1%) | 26 (36.6%) | <0.001 |
| Fine motor | 3,575 (5.7%) | 67 (9.0%) | 17 (18.9%) | 18 (25.4%) | <0.001 |
| Problem solving | 3,150 (5.0%) | 55 (7.4%) | 15 (16.7%) | 18 (25.4%) | <0.001 |
| Personal-social | 690 (1.1%) | 16 (2.1%) | 1 (1.1%) | 6 (8.5%) | <0.001 |

Data are n (%) unless otherwise specified.

Abbreviations: J-ASQ-3, Japanese translation of the Ages and Stages Questionnaire-Third Edition.

The cutoff scores of the J-ASQ-3 reported for Japanese children are shown in Additional file 4. ^†^ Cochran-Armitage test.
