# Supplementary material for: Association between surgical procedures under general anesthesia in infancy and developmental outcomes at 1 year: the Japan Environment and Children’s Study
Source: Environ Health Prev Med. 2020 Jul 25;25:32. doi: 10.1186/s12199-020-00873-6 (PMC7382792; doi:10.1186/s12199-020-00873-6)
Supplement: Supplementary file 6 — Additional file 6. Adjusted odds ratios of developmental delay among infants who had surgical procedures under general anesthesia compared with infants who did not have surgery, for each of the five domains using the cutoff scores of the J-ASQ-3 reported for Japanese children (N = 60,699) [file 12199_2020_873_MOESM6_ESM.docx]

**Additional file 6**  Adjusted odds ratios of developmental delay among infants who had surgical procedures under general anesthesia compared with infants who did not have surgery, for each of the five domains using the cutoff scores of the J-ASQ-3 reported for Japanese children (N=60,699).

|  | Surgery under general anesthesia | | |
| --- | --- | --- | --- |
| J-ASQ-3 | 1 time | 2 times | ≥3 times |
|  | aOR (95% CI) | aOR (95% CI) | aOR (95% CI) |
| Communication | 1.40 (0.30-6.59) | 19.95 (5.98-66.56) * | |
| Gross motor | 1.71 (1.31-2.24) | 5.27 (3.21-8.68) | 7.92 (4.63-13.54) |
| Fine motor | 1.17 (0.88-1.56) | 2.57 (1.45-4.56) | 4.85 (2.69-8.73) |
| Problem solving | 1.18 (0.86-1.62) | 2.62 (1.44-4.78) | 5.98 (3.35-10.67) |
| Personal-social | 1.44 (0.80-2.60) | 2.96 (1.25-6.98) * | |

Abbreviations: aOR, adjusted odds ratio; CI, confidence interval; J-ASQ-3, Japanese translation of the Ages and Stages Questionnaire-Third Edition. Adjusted for sex, gestational age, birth weight, Apgar score at five minutes, delivery method, maternal age at birth (analyzed as a continuous variable), presence of siblings, presence of congenital disease, compared with infants who did not have surgery.

*As for the communication and personal-social domains, the analyses were performed by combining the infants who had surgical procedures twice and three times or more, because there were few infants with developmental delay who had undergone operation twice or more.

The cutoff scores for the J-ASQ-3 reported for Japanese children are shown in Additional file 4.
